# Supplementary figures and images for: A spectral-domain optical coherence tomographic analysis of Rdh5-/- mice retina
Source: PLoS One. 2020 Apr 9;15(4):e0231220. doi: 10.1371/journal.pone.0231220 (PMC7144952; doi:10.1371/journal.pone.0231220)

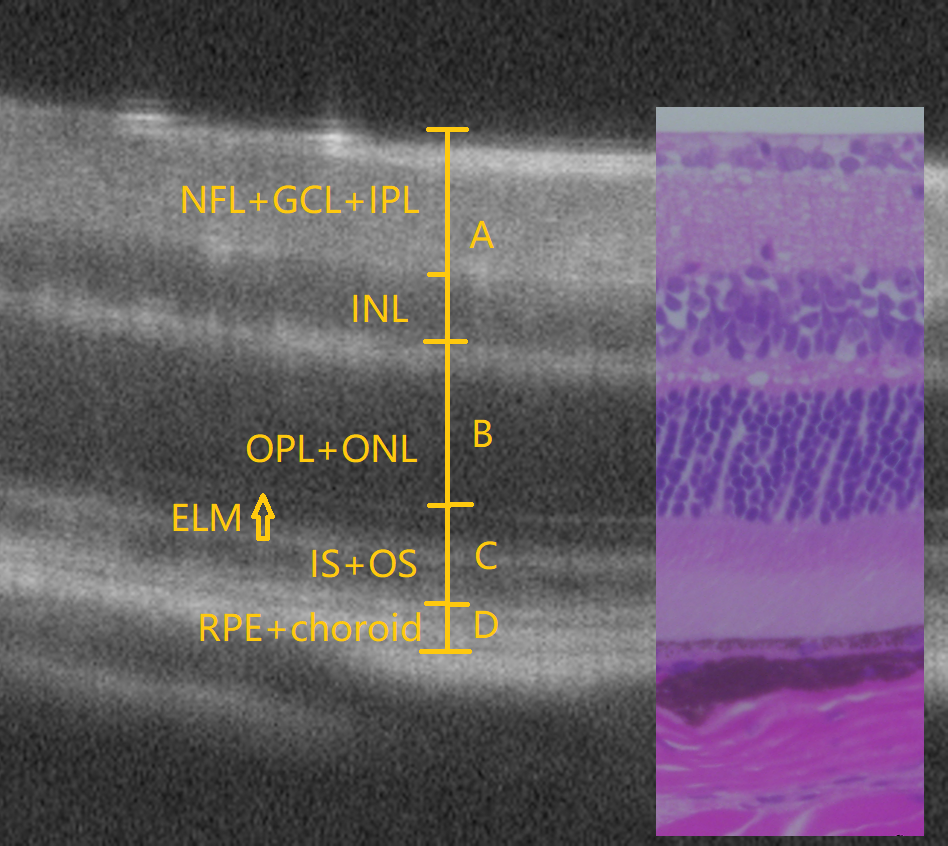

Supplement: S1 Fig — Definition of retinal sublayers A, B, C and D, ELM, IS-EZ and IZ, and comparison between a representative OCT image and histological findings of an Rdh5-/- mouse at PM3. Abbreviations: NFL, nerve fiber layer; GCL, ganglion cell layer; IPL, inner plexiform layer; INL, inner nuclear layer; OPL, outer plexiform layer; ONL, outer nuclear layer; ELM, external limiting membrane; IS-EZ, inner segment ellipsoid zone; IZ, interdigitation zone; RPE, retinal pigment epithelium. (TIF) [file pone.0231220.s001.tif]
