# Supplementary material for: A spectral-domain optical coherence tomographic analysis of Rdh5-/- mice retina
Source: PLoS One. 2020 Apr 9;15(4):e0231220. doi: 10.1371/journal.pone.0231220 (PMC7144952; doi:10.1371/journal.pone.0231220)
Supplement: S1 Table — Raw data for the retinal layer analysis (μm) in C57BL/6J mice. (PDF) [file pone.0231220.s002.pdf]

Retinal layer thickness of C57BL/6J

Age (PM) number	Inner Retinal Layer (A) NFL, GCL, IPL, INL	Outer Retinal Layer (B) OPL, ONL	IS/OS Layer (C) IS, OS	RPE + choroid Layer (D) RPE, choroid
PM1				
1	91.441	70.589	33.066	31.225
2	93.521	64.051	39.807	29.978
3	88.046	66.351	40.361	33.228
4	89.376	63.997	39.95	38.493
mean ± SD	90.596 ± 2.399	66.247 ± 3.096	38.296 ± 3.495	33.231 ± 3.755
PM2				
1	82.673	68.891	44.821	35.491
2	84.091	63.793	42.846	30.9
3	86.075	64.615	37.319	30.512
4	88.656	66.394	36.139	36.212
mean ± SD	85.374 ± 2.595	65.923 ± 2.257	40.281 ± 4.281	33.279 ± 2.990
PM3				
1	86.352	66.668	36.347	36.96
2	92.014	69.398	47.181	34.945
3	109.557	66.947	42.419	33.532
4	87.982	67.253	41.051	31.859
mean ± SD	93.976 ± 10.656	67.567 ± 1.244	41.750 ± 4.458	34.324 ± 2.163
PM4				
1	90.025	71.851	47.854	29.422
2	87.359	66.825	44.644	33.327
3	85.157	68.026	43.692	33.11
4	85.966	68.067	43.588	37.506
mean ± SD	87.127 ± 2.136	68.692 ± 2.183	44.945 ± 1.997	33.341 ± 3.305
PM5				
1	89.052	68.679	39.985	36.087
2	94.119	72.767	45.038	35.207
3	89.143	65.654	41.136	38.892
4	90.117	68.214	42.643	37.452
5	91.244	70.877	48.61	34.218
6	95.022	70.374	46.998	34.819
mean ± SD	91.450 ± 2.560	69.428 ± 2.467	44.068 ± 3.390	36.113 ± 1.767
PM6				
1	88.425	70.035	44.885	35.125
2	88.085	64.418	40.574	34.296
3	89.597	66.761	41.869	35.384
4	89.527	71.183	43.915	36.002
5	84.874	65.449	41.08	33.952
6	90.64	69.361	41.989	38.006
mean ± SD	88.525 ± 2.008	67.868 ± 2.716	42.385 ± 1.673	35.461 ± 1.451
