# Supplementary material for: A spectral-domain optical coherence tomographic analysis of Rdh5-/- mice retina
Source: PLoS One. 2020 Apr 9;15(4):e0231220. doi: 10.1371/journal.pone.0231220 (PMC7144952; doi:10.1371/journal.pone.0231220)
Supplement: S2 Table — Raw data for the retinal layer analysis (μm) in Rdh5−/− mice. (PDF) [file pone.0231220.s003.pdf]

Retinal layer thickness of *Rdh5* -/-

Age (PM) number	Inner Retinal Layer (A) NFL, GCL, IPL, INL	Outer Retinal Layer (B) OPL, ONL	IS/OS Layer (C) IS, OS	RPE + choroid Layer (D) RPE, choroid
PM1				
1	79.426	57.610	35.466	36.223
2	84.458	54.607	38.795	33.197
3	83.194	56.211	35.591	36.913
4	85.334	53.941	38.690	28.638
5	80.789	56.793	34.381	28.903
6	75.686	54.081	33.990	32.007
mean ± SD	81.481 ± 3.604	55.541 ± 1.540	36.152 ± 2.098	32.647 ± 3.515
PM2				
1	83.451	61.407	35.693	36.832
2	83.895	58.619	38.888	32.242
3	81.526	56.524	39.922	29.860
4	81.796	59.320	33.019	34.356
5	77.959	55.550	36.181	28.212
6	78.577	56.402	34.744	33.140
mean ± SD	81.196 ± 2.455	57.970 ± 2.212	36.408 ± 2.581	32.440 ± 3.099
PM3				
1	81.513	56.838	40.441	32.766
2	79.442	54.638	39.648	32.843
3	82.777	57.359	38.980	33.519
4	83.775	57.770	41.678	32.559
5	84.620	55.087	42.913	36.298
6	85.011	53.707	39.985	35.227
mean ± SD	82.856 ± 2.101	55.900 ± 1.647	40.608 ±1.445	33.869 ± 1.540
PM4				
1	95.821	56.427	35.003	30.783
2	92.339	52.093	42.374	29.853
3	82.258	52.746	37.439	25.114
4	80.636	54.141	37.524	23.952
5	78.328	53.339	41.327	27.224
6	77.933	54.164	37.253	33.282
7	81.714	52.327	37.423	25.868
8	84.658	52.580	38.840	32.631
mean ± SD	84.211 ± 6.524	53.477 ± 1.425	38.398 ± 2.392	28.588 ± 3.538
PM5				
1	81.359	54.648	35.715	34.309
2	86.848	48.183	37.351	32.566
3	84.001	49.121	38.206	33.899
4	81.576	50.624	38.382	33.837
mean ± SD	83.446 ± 2.565	50.644 ± 2.852	37.414 ± 1.219	33.653 ± 0.754
PM6				
1	81.12	56.12	35.8	34.51
2	80.58	62.61	42.24	31.14
3	80.46	53.53	40.18	33.6
4	85.52	56	42.16	35.2
5	80.08	57.69	38.84	32.73
6	77.22	56.7	36.7	32.38
mean ± SD	80.829 ± 2.679	57.104 ± 2.101	39.319 ± 2.713	33.258 ± 1.483
