# Supplementary material for: A spectral-domain optical coherence tomographic analysis of Rdh5-/- mice retina
Source: PLoS One. 2020 Apr 9;15(4):e0231220. doi: 10.1371/journal.pone.0231220 (PMC7144952; doi:10.1371/journal.pone.0231220)
Supplement: Attachment — Submitted filename: Responses to Reviewers Comments.docx [file pone.0231220.s004.docx]

Responses to Reviewers’ comments

Reviewer #1

1. Style:
   The notation of protein and gene should name according to the guidelines set by the International Committee on Standardized Genetic Nomenclature for Mice (<https://www.jax.org/jax-mice-and-services/customer-support/technical-support/genetics-and-nomenclature>) both for C57BL6 (e.g. C57BL/6N) and for the mutant.

Responses

We have changed the notation of protein and gene according to the guidelines set by the International Committee on Standardized Genetic Nomenclature for Mice. “*RDH5*^-/-^ mice” has been changed to “*Rdh5*^-/-^ mice” and “C57BL6 mice” has been changed to “C57BL/6J mice”, respectively. In addition, we have added the strain ID for each mouse group like “C57BL/6J (B6)” and “*Rdh5*^-/-^ (B6;129S-Rdh5^tm1Drie^/J)” as recommended by the Committee in the Materials and methods section, Experimental subsection in page 6.

2. In the discussion part the sentence “This may be due to spices-related differences...” should correct to “This may be due to species-related differences...”

Responses

We have corrected the sentence to “This may be due to species-related differences...” in the Discussion section in page 13.

3. Results:
   Fig 2: The wavelength and the orientation of the OCT-scan (horizontal or vertical orientation) should be completed in the legend and/or in the images. The scans show predominantly a wavelike retinal structure. Is this phenomenon based on properties of the device or may due to any phenotype characteristics?

Responses

The orientation of the OCT images has been added as “The position of the retinal SD-OCT image was set circumferentially around the optic disc (360°; diameter, 500 µm; 140 µm away from the optic disc margin, indicated by circles in Fig 1)” in Fig 2 Legend. The wavelike features in some OCT images were artificially made by the different angle between the mouse eye and the eye lens of the OCT apparatus. Therefore, we have added the sentence “The wavelike features in some images were artificially created depended on the angle of the mouse eye against the eye lens” in the Fig 2 Legend. The wavelength of the OCT-scan has been added in the Fig 2 Legend like “The SD-OCT images were made using light stimulation at 830 nm” in page 24. In addition, we have added the sentences “The wavelike features in some OCT images (Fig 2) were artificially created dependent on the angle between the mouse eye and the eye lens attached to the apparatus. Therefore, this phenomenon was not due to any of phenotypic characteristics but was based on properties of the device.” in the Results section in page 10.

4. Fig 3: Please correct the arrow color in C.

Responses

We have changed the arrow color in Fig A and C in black.

5. Fig 5: Please adapt the position of the scale bars as well the size of images on a consistent standard.

Responses

We have changed the position of scales in Fig 5.

6. Fig 7: Please adapt and correct the magnification properties in panel A-E. It appears that panel C shows a higher magnification. Panel F is not included in the Fig but described in the legend. Please correct.

Responses

We have added the explanation of the size of scale bars in the Fig 7 Legend as “Bars indicate 10 µm in panels A, B, D and E, and 2 µm in panel C”.

7. Fig 8: It might be helpful for a better understanding of the ERG recordings if the months of investigation and the species (mutant vs control) are included into the figures.
   Results and discussion:
   The authors have declared no statistical significant differences in the ERG recordings between mutants and controls at PM3 and 5 (Fig 8). But, it might be that the b-wave part in mutations is smaller than in the controls at both time points of investigations by a careful consideration. Therefore, I recommend an overlay of the ERG recordings to proof this suggestion. Furthermore, I recommend to present a separate illustration of the ERG recordings under scotopic (dark adapted), rod-only, as well under scotopic (light adapted), cone-dominated, conditions (please see the publication: Tanimoto et al. 2009, Vision tests in the mouse: Functional phenotyping with electroretinography. Front Biosci (Landmark Ed). Additionally, in 2017, Kuehlewein et al. has described a cone dysfunction in patients with FA.

Responses

As was also pointed out by the reviewer #2, we have recognized that our ERG data were inadequate, because of insufficient number of animals and only a single maximal rod-cone response without any of the other stimulation protocols. Because we are certain that the SD-OCT findings and morphological analyses of the retina in *Rdh5*^-/-^ mice performed in this study are still valuable even without ERG analysis, we took the reviewer #2’s recommendation into account and we have decided that we do not touch the topic of ERG in the manuscript. Therefore, we have deleted the descriptions regarding ERG experiments including Fig 8 from the revised manuscript.

Reviewer #2

1. M&M:
   The authors called the layers of photoreceptor inner segments and outer segments “photoreceptor layer C”, although the photoreceptor cell bodies stretch to the outer rim of the outer plexiform layer, with their nuclei constituting the outer nuclear layer. Maybe the authors could call their layer C “photoreceptor segments layer” or so?

Responses

We have changed the name “photoreceptor layer C” to “photoreceptor segments layer C” throughout the manuscript.

2. Results:
   The authors write: “No qualitative differences were observed in the retina between RDH5-/- and C57BL6 mice. In addition, this tendency was consistent even in the midperipheral areas (Fig 3).” Do they mean Fig. 2 instead of Fig. 3?

Responses

The OCT images in Fig 2 are basically retinal section around the optic disc. Therefore, they do not reflect the findings of the mid-peripheral portion. The OCT images in Figs 3B and 3D partly include the retinal finding in the mid-peripheral portion. Therefore, we mention “No qualitative differences were observed in the retina between RDH5^-/-^ and C57BL6 mice. In addition, this tendency was consistent even in the midperipheral areas (Fig 3).” In order not to make readers confused, we have changed these sentences to “No qualitative differences were observed in the retina between *Rdh5*^-/-^ and C57BL/6J mice (Fig 2). In addition, this tendency was consistent even in the mid-peripheral areas (Figs 3B and 3D).” in page 10.

3. The reviewer has the impression that OCT scans shown in Figs. 3B and 3D do not correspond to the horizontal lines visible in Fig. 3A and 3C. Moreover, it would be nice to have arrows indicating the sites of the hyperreflective spots also in Figs. 3B and 3D.

Responses

Figs 3B and 3D do correspond to the horizontal lines, but orientations are opposite to those of Figs 3A and 3C. This derives from the SD-OCT machine’s property. To make the putative readers correctly understand this relationship, we have added the sentence “Note that the orientations of B and D are opposite to those of A and C.” in Fig 3 Legend. The arrows indicate the whitish spots in the fundus photos not the hyperreflective spots. Because we were unable to detect any hyperreflective spots corresponding to the whitish spots, we do not think that we need to add arrows at the “hyperreflective spots”.

4. In Figure 5, the scale bars are strange, and also the general appearance of the pictures. Were the pictures of the histological sections collected from different sources?

Response

As pointed out by both reviewers, we have corrected the position of scale bars.

5. Discussion:
   In the sentence “This may be due to spices-related difference between mice and humans.” May be a typo.

Responses

As pointed out by both reviewers, we have corrected the typographical error.

6. The biggest problem the reviewer sees in the presented manuscript are the electroretinography (ERG) data that are provided in supplement S4. Firstly, the number of animals (n=4) is by far too small to make any meaningful statements. In general, it is estimated that there may occur a deviation of 10-15% between single ERG measurements. In order to achieve sufficient statistical power to check for differences between experimental groups, approximately 10 animals per group are necessary.
   As the next, it is very questionable to perform a normality test with a group of only n=4. It simply does not make any sense! Anyhow, Shapiro-Wilk normality test is not recommended in many cases, as the D'Agostino & Pearson omnibus normality test is better.
   The deviations inside each group are very big, which raises the question if the ERG measurements were performed properly, and if these ERG data are useful at all. The reviewer has a long history in ERG and VEP measurements in rats and mice, and he finds the big deviations inside the groups very surprising, in particular with respect to the first mouse in the two PM5 groups. Moreover, the big decline of amplitudes in wild-type mice from PM3 to PM5 is surprising and was never observed by the reviewer in his own work. In contrast, almost no amplitude decline was seen in the RDH5-/- mice, which is surprising.
   Consequently, the authors are advised to thoroughly check the ERG data and perform the measurements in a higher number of animals, or to not touch the topic of ERG in their manuscript at all.

Responses

As pointed out by both reviewers, we have realized that the ERG data in this study were quite insufficient because of the comments raised by the reviewer #2. Because it will be difficult for us to further perform ERG assessments so as to fulfill the reviewers’ comments and because we believe that our OCT data with morphological analyses would be still valuable even without ERG analyses, we have decided not to touch the topic of ERG in this manuscript at all, according to the reviewer’s suggestion. Therefore, we have deleted the portions describing ERG from the revised manuscript. In addition to the Shapiro-Wilk test, we also carried out the Kolmogorov-Smirnov test to check the normality of the data.
