# Supplementary material for: A spectral-domain optical coherence tomographic analysis of Rdh5-/- mice retina
Source: PLoS One. 2020 Apr 9;15(4):e0231220. doi: 10.1371/journal.pone.0231220 (PMC7144952; doi:10.1371/journal.pone.0231220)
Supplement: Attachment — Submitted filename: Responses to Reviewers Comments 2.docx [file pone.0231220.s005.docx]

Responses to Reviewers’ comments

Reviewer #1

1. In the discussion part the sentence “This may be due to spices-related differences...” should correct to “This may be due to species-related differences...”

Response:

Thank you very much for detecting the typographical error. We corrected the miss-spelled word “spices” to “species” in the Discussion section.

2. Fig 3: Please correct the arrow colour in C.

Response:

As suggested by the reviewer, we changed the color of the horizontal arrow (line) from green to black in Fig 3.
