# Supplementary material for: A spectral-domain optical coherence tomographic analysis of Rdh5-/- mice retina
Source: PLoS One. 2020 Apr 9;15(4):e0231220. doi: 10.1371/journal.pone.0231220 (PMC7144952; doi:10.1371/journal.pone.0231220)
Supplement: Attachment — Submitted filename: Responses to the editor.docx [file pone.0231220.s006.docx]

Responses to the editor’s comments

Thank you for your valuable comments.

“Why do you think that a pure morphological study describing the retinal changes without discussing the potential effect on the retinal function is sufficient for publication in PONE?”

Response:

Previously, Driessen et al. [16] and Kim et al. [19] have extensively performed ERG experiments in detail. Their results are summarized by the fact that the photoexcitation of the a- and b-waves was not defective in *RDH5^-/-^* mice using standard dark-adapted flash ERG, but the recovery of the a-wave from an intensive bleaching condition was delayed in *RDH5^-/-^* mice compared with that in wild-type mice. Because their experiments were quite extensively and delicately performed, we believe that their results are quite informative and that we do not need to add any further ERG experiments to be performed in this study. The previous reports have detected substantially mild ERG defects in *RDH5^-/-^* mice [16, 19]. We speculate that these mild functional defects may be related with the relatively mild structural changes observed in this study using SD-OCT and electron microscopy. Particularly, we conclude that SD-OCT can quantitatively and non-invasively analyze morphologic changes.

For these reasons, we have changed the paragraph in the Discussion section (P.14) of the previous manuscript, “A study of the photoreceptor function in *Rdh5^-/-^Rdh11^-/-^* mice conducted by Maeda et al. showed that aberrant cone responses were detected until 12-months of age by flicker ERG [41]. Therefore, we speculate that these differences in the metabolisms between mice and humans may be why abnormal findings were hardly detected, at least qualitatively, in the SD-OCT images of *Rdh5^-/-^* mice. In addition, we consider that these metabolic differences between mice and humans may lead to the lack of any marked differences in the amplitudes of ERG a- and b-waves in both *Rdh5^-/-^* and C57BL/6J mice [16, 19] “

to

“Regarding functional aspects, since Driessen et al. [16] and Kim et al. [19] have previously extensively performed electrophysiologic studies in detail, we thought that the results of their studies were quite informative, even though we did not perform electroretinographic (ERG) experiments in this study. Their studies regarding the photoreceptor function in *RDH5^-/-^* mice can be summarized by the fact that the photoexcitation function was not defective in *RDH5^-/-^* mice using standard dark-adapted single flash ERG experiments [16, 19]. However, the recovery of the a-wave from an intensive bleaching condition was delayed in *RDH5^-/-^* mice compared with that in wild-type mice, although no histologic difference was detected between *RDH5^-/-^* and wild-type mice [16, 19]. The results of these previous studies suggested that *RDH5^-/-^* mice showed substantially milder electrophysiological deficits than patients with FA. We speculate that these differences in the electrophysiology may be due to the different metabolisms between mice and humans. In addition, these differences in the metabolisms between mice and humans may be why hardly any abnormal findings were detected, at least qualitatively, in the SD-OCT images of *Rdh5^-/-^* mice. In the revised manuscript (P14, L267 to P15, L281).

In addition, we have added the sentences “However, it is possible that these quantitative changes observed by SD-OCT may be related to the defective photo-recovery function in *RDH5^-/-^* mice [16, 19]. Further studies should be carried out in the future to clarify this point.” in P 15, L280-282 to clarify the ERG findings previously reported.
